# Global transcriptome analysis reveals distinct expression among duplicated genes during sorghum-interaction

**DOI:** 10.1186/1471-2229-12-121

**Published:** 2012-07-29

**Authors:** Hiroshi Mizuno, Hiroyuki Kawahigashi, Yoshihiro Kawahara, Hiroyuki Kanamori, Jun Ogata, Hiroshi Minami, Takeshi Itoh, Takashi Matsumoto

**Affiliations:** 1 National Institute of Agrobiological Sciences (NIAS), Agrogenomics Research Center, 1-2, Kannondai 2-chome, Tsukuba, Ibaraki 305-8602, Japan; 2 Mitsubishi Space Software Co. Ltd, Takezono 1-6-1, Tsukuba, Ibaraki 305-0032, Japan

## Abstract

**Background:**

Sorghum (*Sorghum bicolor* L. Moench) is a rich source of natural phytochemicals. We performed massive parallel sequencing of mRNA to identify differentially expressed genes after sorghum BTx623 had been infected with *Bipolaris sorghicola*, a necrotrophic fungus causing a sorghum disease called target leaf spot.

**Result:**

Seventy-six-base-pair reads from mRNAs of mock- or pathogen-infected leaves were sequenced. Unannotated transcripts were predicted on the basis of the piling-up of mapped short reads. Differentially expressed genes were identified statistically; particular genes in tandemly duplicated putative paralogs were highly upregulated. Pathogen infection activated the glyoxylate shunt in the TCA cycle; this changes the role of the TCA cycle from energy production to synthesis of cell components. The secondary metabolic pathways of phytoalexin synthesis and of sulfur-dependent detoxification were activated by upregulation of the genes encoding amino acid metabolizing enzymes located at the branch point between primary and secondary metabolism. Coordinated gene expression could guide the metabolic pathway for accumulation of the sorghum-specific phytochemicals 3-deoxyanthocyanidin and dhurrin. Key enzymes for synthesizing these sorghum-specific phytochemicals were not found in the corresponding region of the rice genome.

**Conclusion:**

Pathogen infection dramatically changed the expression of particular paralogs that putatively encode enzymes involved in the sorghum-specific metabolic network.

## Background

Plants synthesize low-molecular-weight phytoalexins via secondary metabolic pathways to protect themselves from pathogens such as fungi [1]. Phytoalexins of sorghum such as 3-deoxyanthocyanidins first appear in the cells under fungal attack, where they accumulate in cytoplasmic inclusion bodies. The inclusions migrate to the site of attempted penetration, become pigmented, and ultimately release phytoalexins to kill the fungus [2]. Phytoalexins are produced mainly from aromatic amino acids (phenylalanine (Phe), tyrosine (Tyr)) by the action of many enzymes that sequentially catalyze biochemical reactions. Phenylalanine ammonia lyase (PAL) catalyzes the deamination of Phe to *trans*-cinnamic acid; this is the first step in the biosynthesis of various phenylpropanoids, coumarins, flavonoids, and lignin [3-5]. Aromatic-L-amino acid decarboxylase catalyzes decarboxylation of Tyr to tyramine; this is the first step in the production of isoquinoline alkaloids. Cytochrome P450s are diversified to make various phytoalexins and participants in metabolic networks, such as anthocyanins, tannins, flavones, and isoflavonoid [6,7]. For phytoalexin synthesis, proper enzyme activity is required not only to synthesize the products needed, but also to avoid the accumulation of toxic metabolites.

Sorghum (*Sorghum bicolor* L. Moench) is the fifth most commonly grown cereal in the world and is a rich source of sorghum-specific natural products. In response to pathogen infection, sorghum synthesizes a unique class of flavonoid phytoalexins, namely 3-deoxyanthocyanidins [2,8]. These are structurally similar to anthocyanins except that they lack C-3 hydroxylation. As another example, sorghum seedlings accumulate high levels of dhurrin, a cyanogenic glycoside derived from tyrosine [9]. Degradation of dhurrin releases hydrogen cyanide (HCN), which is very toxic to animals, plants, insects, and microorganisms [10,11]. Dhurrin is also biologically important as a nitrogen storage compound. [12,13]. Sorghum roots exude sorgoleone, a hydrophobic *p*-benzoquinone compound that inhibits electron transfer in photosystem II to preclude competition for resources with neighboring plants [14]. However, the enzymes required for phytoalexin synthesis have not been fully identified, and the nature of the coordinated gene expression for the production of these enzymes remains to be elucidated.

Target leaf spot is one of the major foliar diseases of sorghum under conditions of high humidity. This diseaseis caused by a necrotrophic fungus, *Bipolaris sorghicola*[15,16]. Infected leaves of BTx623 have usually orange to red spots with straw-colored centers. Target leaf spot substantially reduces the production of plant biomass.

Studies of the functional genomics of sorghum began only after completion of the genomic sequence of sorghum BTx623 in 2009 [17]. Sorghum has many proximally duplicated genes. For example, genes encoding cytochrome P450 enzymes are abundant in sorghum, with 326 copies, including the longest tandem gene array, of 15 genes [17]. Each gene may be expressed under the conditions appropriate for catalyzing a particular biochemical reaction. However, the similarity of these genomic sequences makes it difficult to distinguish the expression of gene members of this family, even though various applications have been developed for detecting SNPs by using real-time quantitative reverse transcription – polymerase chain reaction (qRT-PCR) or microarray technology. Moreover, computational annotation has not yet fully covered whole genes. It is therefore important to identify whole transcripts (including unannotated transcripts) for complete gene expression profiling, and there is a need to develop technologies beyond arrays. Given the rapid progress of massive parallel sequencing technology, whole mRNA sequencing (mRNA-seq) has been used for gene expression profiling [18-22]. A series of programs have been developed for building gene models directly based on the piling-up of short reads: the program Bowtie efficiently maps short reads on genomic sequences [23], TopHat concatenates adjacent exons and identifies reads that bridge exon junctions [24], and Cufflinks [25] constructs gene models on the basis of the exons and bridging sequences predicted by Bowtie and TopHat. Thus, the use of sequencing-based expression profiling has the potential to overcome the limitations of PCR- or array-based profiling and can be used to identify key genes expressed among family members.

Here, from among duplicated genes we aimed to identify the key genes required for phytoalexin synthesis and to elucidate their coordinated expression in sorghum after infection with *Bipolaris sorghicola*. For this purpose, we performed whole mRNA sequencing by using massive parallel sequencing technology; differentially expressed genes, including unannotated genes, were identified on the basis of the piling-up of mapped reads. The differentially expressed genes were mapped on metabolic pathways; this analysis revealed their coordinated expression in primary metabolic networks to change the role of the TCA cycle and amino acids. We compared the expression of these genes with those of tandemly duplicated family genes in the sorghum genome and identified key enzymes in sorghum-specific phytoalexin synthesis. We also compared the genes with those located in the corresponding genomic regions in rice, and we discuss the evolutionary history of sorghum-specific phytoalexin synthesis. This work will help to elucidate the transcriptional regulation of primary and secondary metabolic pathways in response to pathogen infection in sorghum.

## Results

### Identification of differentially expressed genes by mRNA-seq

We performed sorghum transcriptome analysis by mRNA-seq. After infection with conidia of *Bipolaris sorghicola,* sorghum BTx623 exhibites typical leaf lesions, which are reddish orange. Pathogen-treated or control (mock-infected) sorghum leaves were collected, and 76-base-pair (bp) reads from mRNAs were sequenced by using Illumina mRNA-Seq technology. Of the 28 to 34 million quality-evaluated reads, a total of 81.7% (infected) or 81.8% (mock-infected) were mapped: 68.0% and 67.8%, respectively, were mapped uniquely to the sorghum genome; 8.5% and 8.7% bridged flanking exons uniquely; and others were mapped to multiple loci (Table 1). This left 18.3% (infected) and 18.2% (mock-infected) unmapped on the sorghum genome. As the ratios of unmapped reads were almost the same in the control and infected samples, we considered that few reads were derived from fungal transcripts.

**Table 1:** Numbers of mapped reads

**Sample**	**Total reads (%)**	**Total mapped (%)**	**Mapped on genome**		**Mapped on junction**		**Total unmapped (%)**
			**Unique (%)**	**Multiple (%)**	**Unique (%)**	**Multiple (%)**	
control	34,569,214	28,260,814	23,433,563	1,753,559	2,995,861	77,831	6,308,400
	100	81.8	67.8	5.1	8.7	0.2	18.2
infected	28,695,822	23,451,544	19,501,177	1,448,478	2,437,660	64,229	5,244,278
	100	81.7	68.0	5.0	8.5	0.2	18.3

Numbers of reads and percentages of the total reads (Total reads) are shown, as are the total numbers of reads mapped (Total mapped), reads mapped uniquely to the sorghum genome (Mapped on genome – unique), reads mapped to multiple loci of the sorghum genome (Mapped on genome – multiple), reads mapped uniquely to a predicted exon–exon bridging sequence (Mapped on junction – unique), reads mapped to multiple loci of the exon–exon bridging sequences (Mapped on junction – multiple), and reads unable to be mapped (Total unmapped).

**Figure 1:**
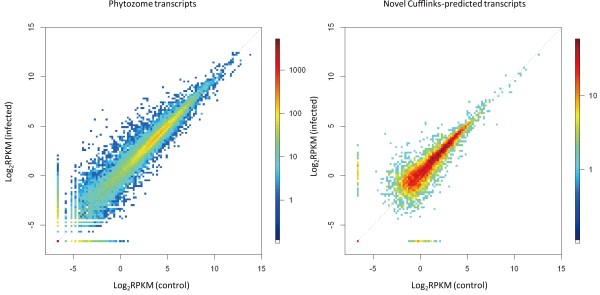
**Comparison of RPKM of each gene after pathogen infection.** RPKM (Reads Per Kilobase of exon model per Million mapped reads) values for Phytozome annotated transcripts (left) or novel Cufflinks-predicted transcripts (right) were compared in mock-infected (control) and pathogen-infected leaves. For each gene, the RPKM (log_2_) value in mock-infected plants is plotted on the horizontal axis and the corresponding RPKM (log_2_) value in pathogen-infected plants is plotted on the vertical axis. Distributions of the number of transcripts are given as colors. A constant of 0.01 was added to each RPKM to avoid zero scores in log calculations.

mRNA-seq quantifies transcripts on the basis of the number of sequence reads mapped on each gene. We adopted RPKM (Reads Per Kilobase of exon model per Million mapped reads)[26] for transcript quantification, and in control and infected leaves we compared the RPKM of each gene annotated in Phytozome (http://www.phytozome.net/; Figure 1); we cited the annotations in Phytozome and searched for the best BLASTx in *Arabidopsis thaliana* (Additional File 1: Table S1). Differentially expressed genes were identified statistically by using the G-test with a 1% false discovery rate (FDR); 5617 transcripts at 5095 loci were upregulated, whereas 3052 transcripts at 2688 loci were downregulated (Table 2). 

**Table 2:** Numbers of differentially expressed genes

		**No. annotated (%)**	**No. of novel (%)**
up	transcripts	5617 (19.1)	816 (10.6)
	loci	5095 (18.5)	594 (9.8)
down	transcripts	3052 (10.4)	239 (3.1)
	loci	2688 (9.7)	196 (3.2)

Numbers of differentially expressed genes and percentages among Phytozome annotations (Sbicolor_79; 29,448 transcripts at 27,608 loci) or novel Cufflinks-predicted transcripts (7674 transcripts at 6063 loci) are shown.

### Constructing gene models and searching for homology to genes encoding known proteins

**Figure 2:**
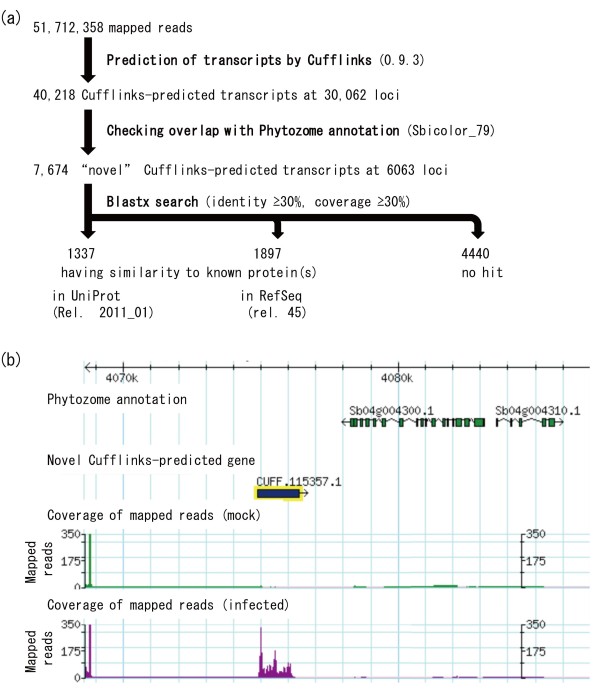
**Strategy for characterizing unannotated transcripts.** (**a**) Novel transcripts predicted on the basis of the piling-up of mapped short reads by using the Cufflinks program. Unannotated transcripts were selected by checking overlap with Phytozome annotation (Sbicolor_79). To predict the functions of the unannotated transcripts, BLASTx searches were performed against known protein datasets, namely Uniprot (Rel. 2011_01) and RefSeq (release 45). (**b**) Differential expression of Cufflinks-predicted transcripts. The graph indicates the coverage of mapped reads from mock- (mock; green), or pathogen-infected (infected; purple) leaves, in GBrowse. Exon models were predicted by the piling-up of reads by the Cufflinks program (Novel Cufflinks-predicted gene; blue box). Gene models annotated in Phytozome are also shown (Phytozome annotation; green boxes). CUFF115357.1 was unannotated and was expressed differentially.

Novel transcripts were identified on the basis of the piling-up of mapped short reads, through the series of programs Bowtie [23], TopHat [24], and Cufflinks [25]. By using 51,712,358 mapped reads (summing the 28,260,814 from the controls and 23,451,544 from the infected tissues; Table 1; Total mapped), 40,218 transcripts at 30,062 loci were predicted (Figure 2A). Checking for overlap with the Phytozome annotation revealed that 7674 transcripts at 6063 loci were unannotated (Figure 2A). To predict the functions of the unannotated transcripts, we performed a BLASTx search against two known protein datasets: 1337 transcripts had similarity (identity ≥30% and coverage ≥30%) to genes encoding known proteins in Uniprot (Rel. 2011_01), and 1897 transcripts had similarity to those in RefSeq (release 45); thus 42.1% (3234/7674) novel transcripts had similarity to known proteins (Figure 2A). In response to pathogen infection, 816 unannotated transcripts at 594 loci were upregulated and 239 transcripts at 196 loci were downregulated (Table 2). The RPKMs of Cufflinks-predicted genes were compared (Figure 1) and differentially expressed genes were identified (Additional File 2: Table S2). Some differentially expressed unannotated transcripts were putatively associated with secondary metabolic pathways of phytoalexin synthesis: CUFF.23467.1 had similarity to maize *ZRP4 o*-methyltransferase [27], and CUFF.115357.1 had similarity to dihydroflavonol 4-reductase (DFR), which catalyzes reduction of the C-4 carbonyl group of naringenin in sorghum [28] (Figure 2B; Additional File 3: Table S3). 

### Characterization of differentially expressed genes

We focused here on primary or secondary metabolic pathways containing proteins encoded by highly differentially expressed genes; we also compared the expression of these genes with those of their family genes. Fifty genes that were extremely differentially expressed were listed (Additional File 4: Table S4).

### Primary metabolism

#### Glyoxylate shunt in the TCA cycle: isocitrate lyase and malate synthase

**Figure 3:**
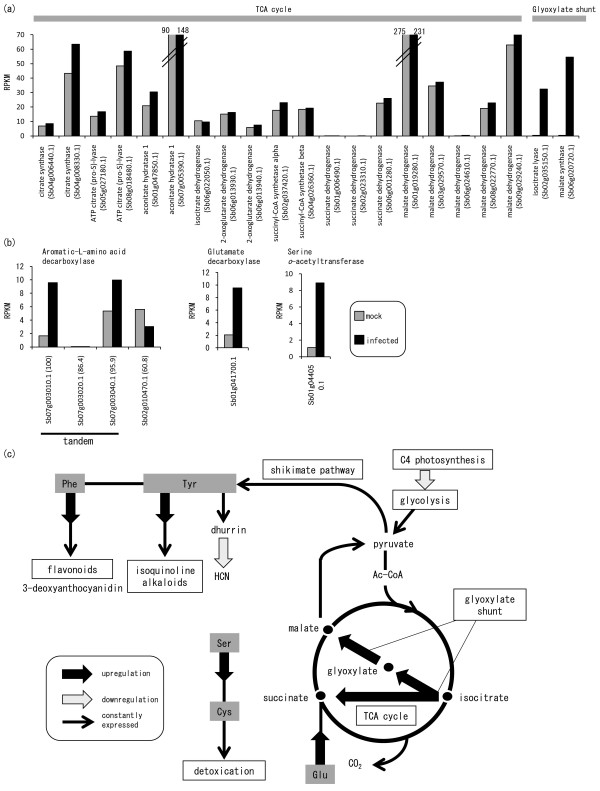
**Changing the role of the TCA cycle and of amino acid metabolism.** (**a**) Expression of genes in the TCA cycle, including the glyoxylate shunt. RPKMs of each gene were compared in mock- (gray bars) and pathogen-infected (black bars) leaves. (**b**) Expression of genes for amino acid metabolism. RPKMs of each gene were compared as in (**a**). (**c**) Roles of glyoxylate shunt and amino acid metabolism in the defense response. Upregulation or downregulation of genes is shown on the metabolic map. The glyoxylate shunt pathway of the TCA cycle skips the CO_2_-generating steps. Subsequently, phenylalanine [Phe] and tyrosine [Tyr] are synthesized through the shikimate pathway. Phe and Tyr are precursors of various flavonoids and isoquinone alkaloids, respectively. Tyr is also a precursor of the sorghum-specific cyanogenic glycoside, dhurrin. Succinate is suppied from glutamate (Glu). Cysteine (Cys) is suppied from Serine (Ser); Cys serves as a precursor for various sulfur-dependent detoxication.

Genes encoding isocitrate lyase (Sb02g035150.1) and malate synthase (Sb06g020720) were highly differentially expressed (86.5 fold and 131.5 fold respectively; Figure 3A and Additional File 4: Table S4). These enzymes are involved in the shunt pathway of the TCA cycle, namely the glyoxylate cycle. Isocitrate lyase cleaves isocitrate to form glyoxylate and succinate, and malate synthase converts glyoxylate and acetyl-CoA to malate. Succinate is used directly in the TCA cycle, and glyoxylate can be used in the TCA cycle after its conversion to malate by malate synthase. As other genes associated with the TCA cycle were not differentially expressed, it appears that only the glyoxylate shunt portion of the cycle was enhanced (Figures 3A and 3C).

#### Amino acid metabolism

Genes encoding aromatic L-amino acid decarboxylase (Sb07g003010) are tandemly duplicated in the sorghum genome (Sb07g003010, Sb07g003020, Sb07g003040), but only Sb07g003010 was highly upregulated in infected leaves (Figure 3B). Aromatic L-amino acid decarboxylase catalyzes L-tyrosine and/or L-tryptophan decarboxylation irreversibly and is responsible for the commitment of aromatic L-amino acids to secondary metabolic pathways such as the isoquinoline alkaloid biosynthesis pathway (Figure 3C).

The gene encoding serine o-acetyltransferase (01 g044050) was induced in infected leaves (Figure 3B); this enzyme catalyzes the formation of *O*-acetyl-Ser from Ser and acetyl-CoA. Subsequently, Cys is formed by the condensation of sulfide and *O*-acetyl-Ser; this is catalyzed by *O*-acetyl-Ser (thiol) lyase [29]. These two steps link Ser irreversibly to Cys biosynthesis (Figure 3C).

The gene encoding glutamate decarboxylase (Sb01g041700) was also induced in infected leaves (Figure 3B); this enzyme catalyses irreversible decarboxylation of glutamate to produce succinate finally, which is supplied to the TCA cycle (Figure 3C).

### Secondary metabolism

Sorghum is a rich source of sorghum-specific phytochemicals, including certain 3-deoxyanthocyanidins [2], dhurrin [13], and sorgoleone [30]. We focused here on differentially expressed genes associated with the synthesis of these sorghum-specific phytochemicals.

#### 3-deoxyanthocyanidin

**Figure 4:**
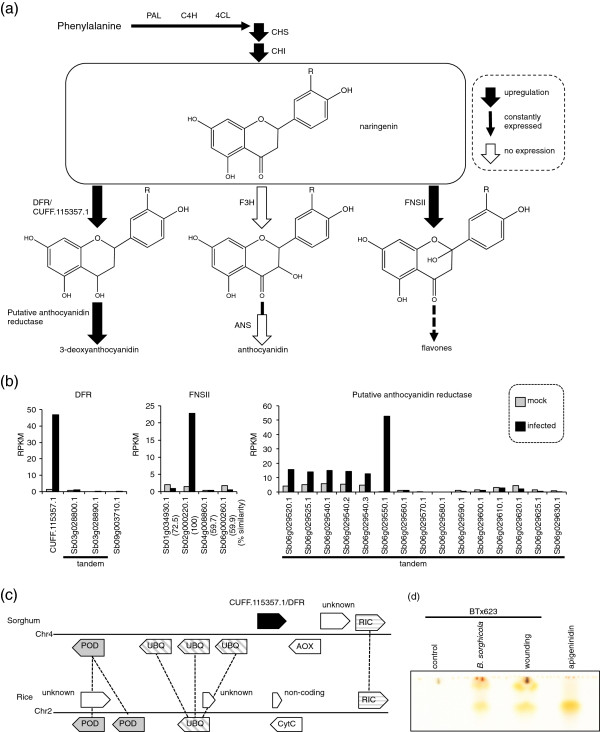
**Biosynthesis of secondary metabolites from phenylalanine.** (**a**) Schematic view of metabolic pathway of 3-deoxyanthocyanidin. Naringenin (center) synthesis from Phe occurs through sequential reactions catalyzed by phenylalanine ammonia lyase (PAL), trans-cinnamate 4-monooxygenase (C4H), 4-coumarate:CoA ligase (4CL), chalcone synthase (CHS), and chalcone isomerase (CHI). 3-deoxyanthocyanidin is synthesized by the action of dihydroflavonol 4-reductase (DFR) and a putative anthocyanidin reductase. Anthocyanidin is synthesized by the actions of flavanone 3-hydroxylases (F3H), and anthocyanindin synthase (ANS) from naringenin. Upregulation of *DFR* and anthocyanidin reductase genes, and suppression of F3H and ANS genes suggests that 3-deoxyanthocyanidin accumulates but anthocyanin does not. FNSII converts naringenin to flavone through the formation of 2-hydroxyflavanones. (**b**) Expression of genes associated with secondary metabolism from naringenin. RPKMs for each gene were compared in mock-infected (gray bars) and pathogen-infected (black bars) leaves. An unannotated gene, CUFF115357.1, was upregulated among four DFR genes. A putative anthocyanidin reductase gene (Sb06g029550) was highly upregulated among tandemly duplicated putative paralogs. Expression of an FNSII gene (Sb02g000220.1) was induced among four family members. (**c**) CUFF115357.1 gene in sorghum and the corresponding region in rice. CUFF115357.1 in sorghum (black) had no corresponding gene in rice. Genes putatively encoded polyubiquitin (UBQ), peroxidase (POD), ROP interacting CRIB motif protein (RIC), amine oxidase (AOX), cytochrome C (CytC), or an unknown protein, or were non-coding transcripts. Corresponding genes are connected by lines. (**d**) Accumulation of apigeninidin after infection. Pigments extracted from leaf of sorghum BTx623 (control, after infection with *Bipolaris sorghicola,* or wounding) were subjected to thin layer chromatography. Chemically synthesized apigeninidin was used as a standard.

Sorghum BTx623 accumulates 3-deoxyanthocyanidin from Phe through naringenin as a common intermediate of anthocyanidin (Figure 4A). 3-deoxyanthocyanin biosynthesis from Phe occurs through sequential reactions catalyzed by phenylalanine ammonia lyase (PAL), trans-cinnamate 4-monooxygenase (C4H), 4-coumarate:CoA ligase (4CL), chalcone synthase (CHS), chalcone isomerase (CHI), dihydroflavonol 4-reductase (DFR), and an unknown anthocyanidin reductase.

PAL is the first enzyme committed to the secondary metabolic pathway that converts Phe to 4-coumaroyl-CoA, the precursor of various phytochemicals. There were six tandemly duplicated sorghum PAL genes on chromosome 4 (Sb04g026510 to Sb04g026560); the amino acids encoded by these genes shared 81.8% or more identity (Sb04g026520 = 100%) (Additional File 5: Figure S1). Some genes encoding PAL, C4H, and 4CL were highly expressed, but they were not differentially expressed after pathogen infection (Additional File 5: Figure S1).

The *CHS* genes are tandemly duplicated (9 genes; Sb05g020150 to Sb05g020230); six were upregulated in infected leaves and four were barely expressed (Additional File 5: Figure S1). *CHI* have three copies dispersed in different chromosomes, and two of them were upregulated (Additional File 5: Figure S1). Upregulation of the genes encoding CHS and CHI suggests the synthesis of naringenin, the precursor of various phytochemicals (Figure 4A).

The DFR genes consisted of four putative paralogs; three were currently annotated and one was predicted on the basis of the piling-up of mapped reads by using the Cufflinks program (Additional File 3: Table S3). The unannotated one, *CUFF.115357.1*, was the only one upregulated (Figures 2B and 4B), suggesting that CUFF.115357.1 is responsible for reduction of the C-4 carbonyl group of naringenin. Part of *CUFF.115357.1* was the same as the sequence previously named *DFR3*[28], but it was not annotated in the latest sorghum annotation (Sbi_79) (Figure 2B). *CUFF115357.1* was located between polyubiquitin genes (UBQ) and the ROP interacting CRIB (Cdc42/Rac-interactive binding) motif protein (RIC) on chromosome 4, and the syntenic region in rice chromosome 2 was identified by aligning the genome sequence with the rice genome (International Rice Genome Sequencing Project Build 5.0 pseudomolecules). However, rice had no *DFR* gene in the corresponding region on chromosome 2 (Figure 4C).

Anthocyanidin reductase, which may be responsible for the subsequent conversion of flavan-4-ols to 3-deoxyanthocyanidins, has not been characterized. We found that Sb06g029550, which is similar to anthocyanidin reductase, was extremely differentially expressed in infected leaves (330.4 fold; Additional File 4: Table S4). Sb06g029550 was the most highly differentially expressed gene among 58 homologous genes, including the 15 tandemly duplicated (Sb06g029510 to Sb06g029630) genes, in BTx623 (Figure 4B). Sb06g029550 had similarity to the *A. thaliana BANYULS* (*BAN*) gene encoding anthocyanidin reductase (Additional File 4: Table S4) [31]. We consider that Sb06g029550 is the candidate responsible for the final step of synthesizing 3-deoxyanthocyanidin. Accumulation of apigeninidin, one of the 3-deoxyanthocyanidins, was detected after infection with *Bipolaris sorghicola* by using thin layer chromatography (TLC) (Figure 4d). Thus, upregulation of *CHSCHICUFF.115357.1/DFR*, and the putative anthocyanidin reductase gene Sb06g029550 suggests the synthesis of 3-deoxyanthocyanidin derived from phenylalanine.

In contrast, genes for anthocyanidin synthesis were barely expressed in BTx623. The sorghum flavanone 3-hydroxylases *F3H1* (Sb06g031790.1) and *F3H2* (unannotated in Phytozome) [28] were not expressed at all (Additional File 1: Table S1), suggesting the blocking of C-3 hydroxylation of naringenin; this blocking is therefore the critical determinant of the production of 3-deoxyanthocyanidin instead of anthocyanidin (Figure 4A). The gene encoding anthocyanidin synthase (ANS; Sb04g000260.1), which catalyzes a downstream reaction for anthocyanidin formation [28,32] was not expressed under either of the conditions studied (Additional File 1: Table S1). Thus, the lack of expression of the F3H and ANS genes supports the accumulation of 3-deoxyanthocyanidin, and not anthocyanidin, in BTx623 (Figure 4A).

The FNSII gene Sb02g000220.1 was upregulated among four putative paralogs (Figure 4B). FNSII is a cytochrome P450 protein (CYP93G3) that converts flavanones (naringenin) to flavones through the formation of 2-hydroxyflavanones (Figure 4A) [33].

Among tandemly repeated family members, particular genes of the F3′H and polyphenol oxidase families were differentially expressed (Additional File 5: Figure S2). The differentially expressed F3’H gene (Sb04g024710.1) was previously named SbF3’H2 which is involved in pathogen-specific 3-deoxyanthocyanidin synthesis (Shih et al.. 2006 ). Putative substrates for sorghum F3’H proteins are naringenin (Shih et al.. 2006) or precursors of flavonoid biosynthesis such as kaempferol (Boddu et al. 2004). The enzymes encoded by polyphenol oxidase genes might be related to phytoalexin synthesis, even though their targets are unknown.

#### Dhurrin

**Figure 5:**
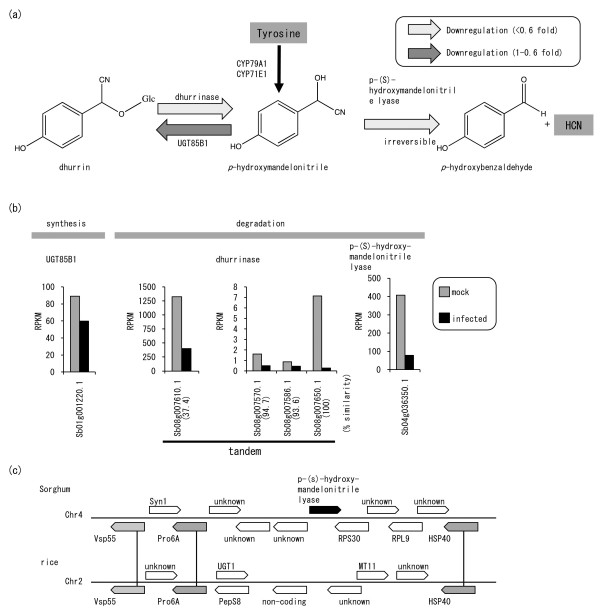
**Synthesis and degradation of dhurrin.** (**a**) Pathway of synthesis and degradation of dhurrin. Dhurrin is synthesized from tyrosine through a p-hydroxymandelonitrile intermediate; this is catalyzed by CYP79A1 and CYP71E1. Subsequently, dhurrin content is regulated in synthesis and degradation. In the synthesis pathway, the UDP-Glc p-hydroxymandelonitrile glycosyltransferase UGT85B1 converts p-hydroxymandelonitrile into dhurrin. In the degradation pathway, dhurrinase and p-(S)-hydroxymandelonitrile lyase sequentially degrade dhurrin and release hydrogen cyanide (HCN) irreversibly. (**b**) Downregulation of genes for degradation of dhurrin. RPKMs for each gene were compared in mock-infected (gray bars) and pathogen-infected (black bars) leaves. (**c**) Region of the p-hydroxymandelonitrile gene and the corresponding region in rice. Sorghum genes encoding vacuolar protein sorting 55 protein–like protein (Vps55), 26 S protease regulatory subunit 6A (Pro6A), or heat shock protein 40 (HSP40) had corresponding genes on rice chromosome 2. Genes in the rice corresponding region encoded UDP-glycosyltransferase 91D1 (UGT; Os02t08039000), peptidase S8 (PepS8; Os02t0803900), methyltransferase type 11 (MT11; Os02t0804300), or an unknown protein (Os02t0804100, Os02t0804400), or were non-coding (Os02t0804000). Genes neighboring p-(S)-hydroxymandelonitrile lyase in sorghum, encoding synaptobrevin 1(Syn1), 40 S ribosomal protein S30 (RPS30), or 50 S ribosomal protein L9 (RPL9), had no corresponding genes in rice.

Degradation and synthesis of dhurrin are catalyzed by different enzymes. Degradation is catalyzed by dhurrinase and P-(S)-hydroxymandelonitrile lyase (Figure 5A). In sorghum, there are four tandemly arranged dhurrinase genes. Three of these dhurrinase genes (Sb08g007570, Sb08g007586, and Sb08g007650) were tandemly duplicated and highly conserved; their encoded proteins shared at least 93.6% amino acid sequence identity (Figure 5B). The protein encoded by another gene, Sb08g007610, had low identity with Sb08g007650 (37.4%), although Sb08g007610 was proximal to the three putative paralogs, but its basal expression level was higher than those of these other three. Expression of all four dhurrinase genes was downregulated (0.04 to 0.53 fold; Figure 5B; Additional File 1: Table S1). Another gene for degradation of dhurrin, the p-(S)-hydroxymandelonitrile lyase gene (Sb04g036350) was extremely downregulated (0.20 fold; Figure 5B; Additional File 1: Table S1). In contrast, a gene for the biosynthesis of dhurrin, which is catalyzed by UGT85B1 (Sb01g001220) [13] (Figure 5A), was slightly downregulated (0.67 fold; Figure 5B; Additional File 1: Table S1). Thus, degradation was substantially suppressed compared with synthesis, suggesting that pathogen infection is likely to promote the accumulation of dhurrin. 

We compared the genomic regions responsible for dhurrin synthesis in sorghum with the corresponding regions in rice. p-(S)-hydroxymandelonitrile lyase catalyzes the critical step of release of the potent toxin HCN (Figure 5A). The p-(S)-hydroxymandelonitrile lyase gene in sorghum was located between the protease 6A (Pro6A) gene and the heat shock protein 40 (HSP40) gene (Figure 5C). Genes in the corresponding rice region encoded UDP-glycosyltransferase 91D1 (Os02t08039000), peptidase S8 (Os02t0803900), methyltransferase type 11 (Os02t0804300), or an unknown protein (Os02t0804100, Os02t0804400), or were non-coding (Os02t0804000). Thus, the p-(S)-hydroxymandelonitrile lyase gene, which is responsible for the final step of dhurrin degradation and HCN release, was not identified in the rice genome (Figure 5C), supporting the hypothesis that dhurrin is a toxic chemical peculiar to sorghum.

#### Sorgoleone

Sorgoleone is a lipid benzoquinone that is produced only by members of the genus *Sorghum *[34-36] Alkylresorcinol synthases (ARSs) play essential roles in the biosynthesis of sorgoleone, which produces 5-alkylresorcinols, by using medium to long-chain fatty acyl-CoA starter units [37]. ARS genes (Sb08g003170, Sb02g034030, Sb05g022500, Sb05g022510) [38] were not expressed in leaves in this study (Additional File 1: Table S1). As sorgoleone is involved in allelopathy, this gene might be expressed only in the root hairs.

## Discussion

### Transcriptional regulation of the TCA cycle, amino acid metabolism, and photosynthesis

Analysis of the functional genomics of sorghum started only after completion of the genomic sequencing of sorghum BTx623 in 2009 [17]. To identify key expressed genes for sorghum-specific phytoalexin synthesis and elucidate their coordinated expression, we performed whole mRNA sequencing by using massive parallel sequencing technology (Table 1); differentially expressed genes, including unannotated genes, were identified on the basis of the piling-up of mapped reads (Tables 2; Figures 1,2; Additional Files 1,2,3,4: Tables S1-S4). We have validated the differential expression of these annotated or Cufflinks-predicted unannotated genes by using qRT-PCR experiment of biological replicates (Additional File 6: Figure S3).

The glyoxylate shunt in the TCA cycle, which involves the action of isocitrate lyase and malate synthase, was activated (Figure 3A). The shunt pathway of the TCA cycle allows increased production of carbon compounds by bypassing the CO_2_-generating steps of the TCA cycle and contributes to the synthesis of cell components such as cell-wall polysaccharides, nucleotides, and amino acids. Genes in the shikimate pathway are ubiquitously expressed (Additional File 1: Table S1); this reinforces the supply of Phe and Tyr, which are precursors of phytoalexins (Figure 3C). The glyoxylate shunt is widespread in plants, bacteria, and fungi [39]. In plants, the glyoxylate shunt is of primary importance for the growth of plant seedlings; it is involved in the conversion of stored lipids to carbohydrates that serve as primary nutrient sources before photosynthesis [40]. However, synthesis of cellar components results in the consumption of components of the TCA cycle. To compensate for this loss, succinate, a substrate for the TCA cycle, could be supplied from glutamate, because production of glutamate decarboxylase (Sb01g041700), which catalyzes the first step from Glu to succinate, was highly induced (Figure 3B). Thus, boosting of the glyoxylate shunt suggests that there is change in the role of the TCA cycle from energy production to synthesis of cellar components.

Amino acids are not only building blocks of protein; they also serve as biosynthetic precursors for anti-pathogen metabolites. Decarboxylation of Tyr (Figure 3C) is the first step in the production of complex isoquinoline alkaloids, which comprise more than 2500 known compounds found in various plants [41]. The upregulation of genes encoding polyphenol oxidase (Additional File 5: Figure S2) also supports the synthesis of isoquinoline alkaloids (Additional File 5: Figure S2). PALs, which were highly expressed constitutively (Additional File 5: Figure S1), are involved in the first step in the biosynthesis of flavonoids by catalyzing the deamination of Phe (Figure 3C). Ser acetyltransferase links Ser metabolism to Cys biosynthesis (Figure 3C). Cys serves as a precursor for various sulfur-containing metabolites, including glutathione (GSH), cofactors, essential vitamins, and sulfur esters [42-44]. The upregulation of genes encoding glutathione S-transferases (Sb02g003090.1, Sb01g030880.1; Additional File 1: Table S1) also supports the activation of GSH-dependent detoxification.These amino acid metabolizing enzymes are located at the branch point between primary and secondary metabolism, suggesting that their upregulation enables irreversible commitment to the pathway (Figure 3C).

An inverse correlation between photosynthesis- and defense-related gene expression has been observed in the C3 plants tobacco [45] and potato [46]. In contrast, sorghum has genes for C4 photosynthesis [17]; six of seven previously identified C4 photosynthesis genes (two for carbonic anhydrase and one each for malate dehydrogenase, malic enzyme, phosphoenolpyruvate carboxylase, and pyruvate orthophosphate dikinase) were downregulated (Additional File 2: Table S2). Even though the changes were small (0.35 to 0.71 fold; Additional File 2: Table S2), the basal expression levels of photosynthesis genes were high (e.g. the RPKM [mock-infected] for pyruvate orthophosphate dikinase was 3915.65; Additional File 5: Figure S2 and Additional File 1: Table S1), and thus the absolute amounts of transcripts would have changed substantially. This response supports the inverse relationship between C4 photosynthesis- and defense-related gene expression in sorghum.

### Coordinated gene expression for sorghum-specific responses

Our mRNA-seq analysis revealed the transcriptional regulation of key enzymatic steps for synthesizing sorghum-specific phytochemicals. By our genome-wide analysis we also identified candidate genes responsible for the missing steps of sequential reaction that causes the accumulation of phytoalexins. Sorghum BTx623 exhibits typical reddish orange leaf lesions after infection with the conidia of *B. sorghicola*. Apigeninidin, one of the 3-deoxyanthocyanidins, was accumulated after infection with *B. sorghicola* (Figure 4d). 3-deoxyanthocyanidin is also accumulated after infection with *Colletotrichumsublineolum *[2] or *Cochliobolus heterostrophus *[47]. In BTx623 we found coordinated gene expression and suppression of genes; this included the upregulation of CHS, CHI, an unannotated DFR gene (CUFF.115357.1) and a putative anthocyanidinreductase candidate (Sb06g029550), as well as the suppression of F3H and ANS genes (Figure 4A). These findings suggest that accumulation of 3-deoxyanthocyanidin, but not anthocyanidin, occurs upon infection with *B. sorghicola*. In another sorghum accession, DK46, anthocyanin pigment is accumulated through sequential reactions catalyzed by F3H, DFR, and ANS [28], suggesting that expression of the genes encoding these proteins has changed during the history of sorghum breeding.

What controls the coordinated expression of such genes? As a candidate, *Yellow seed1* (*Y1*), which encodes a MYB-type regulatory protein, plays pivotal roles in pericarp pigmentation with 3-deoxyanthocyanidin in seeds of sorghum; deletion of the *Y1* allele in BTx623 produces seeds without these 3-deoxyflavonoid pigments [48]. Expression of a putative flavonoid 3’hydroxylase (F3′H) gene is under the control of the sorghum *Y1* gene in synthesizing 3-deoxyanthocyanidin phytoalexins [49]. *P1*, a *y1* homolog in maize, activates the expression of genes encoding CHS and CHI [50]. In this study, leaf expression of *y1* was completely suppressed with or without infection with *B. sorghicola* (Additional file 1: Table S1), but the genes encoding CHS, CHI, and F3′H were differentially expressed (Additional File 5: Figure S1 and S2). We therefore consider that regulation of phytoalexin synthesis could differ between seed and leaf. Other transcription factors may be responsible for the expression of genes for 3-deoxyanthocyanidin production in the leaves of BTx623. Expression of genes for transcription factor families such as ERF, WRKY, DREB, and the zinc finger family was induced (Additional File 4: Table S4). Transcription factors were also duplicated in the sorghum genome. For example, a number of WRKYs have been annotated and have had a lineage-specific gene expansion during the course of plant evolution: one in *Chlamydomonas reinhardtii*, 37 in the moss *Physcomitrella patens*, 74 in Arabidopsis, almost 200 in soybean, and 93 in sorghum [51,52]. Expansion of the numbers of genes of this family (i.e. WRKY) is likely to be associated with the ongoing development of highly sophisticated defense mechanisms co-evolving in plants together with pathogens.

Dhurrin content could be regulated by changes in both synthesis and degradation (Figure 5A); degradation of dhurrin results in release of HCN, which can be lethal to animals, insects, fungi, and plants [13,53,54]. In the sorghum genome we identified genes responsible for dhurrin metabolism, and we showed that pathogen infection favored the accumulation, not degradation, of dhurrin (Figure 5B). As the release of HCN inhibits phytoalexin production [55], HCN might be more damaging to the plant than to the invader. Therefore, in the case of fungal infection, the genes responsible for dhurrin degradation might be strictly suppressed. Moreover, expression of *CYP79A1*, which is responsible for the synthesis of p-hydroxymandelonitrile (an intermediate of dhurrin; Figure 5A), was also slightly suppressed by infection (Additional File 1: Table S1), whereas *CYP79A1* expression is induced by feeding greenbugs [56]. Thus, the defense mechanism related to dhurrin in fungal infection might differ from that in insect feeding.

### Evolutionary history of phytoalexin synthesis after sorghum and rice split

The size of the sorghum genome is approximately 730 Mb [17], which is twice the 389 Mb of the rice genome [57]. This difference in size is due mainly to differences in the content of repetitive sequences: 55% of the sorghum genome consists of retrotransposon sequences, compared with the smaller rice genome (26%) [17]. Alignment of genetic [58] and cytological maps [59] suggests that sorghum and rice have similar quantities of euchromatin (252 and 309 Mb, respectively), with a largely collinear gene order [60]. Nevertheless, some of the genes in the sorghum genome were duplicated after the sorghum–rice split. We demonstrated the tandemly duplicated genes and the diversity of pathogen-inducible expression of the genes encoding aromatic-L-amino acid decarboxylase (Figure 3B), DFR, putative anthocyanidin reductase (Figure 4B), dhurrinase (Figure 5B), PAL, CHS (Additional File 5: Figure S1), F3′H, and polyphenol oxidase (Additional File 5: Figure S2). The synthesis of sorghum-specific phytochemicals was explained by the presence of the sorghum-specific genes encoding p-(S)-hydroxymandelonitrile lyase and CUFF115357.1/DFR3, which were acquired after the sorghum–rice split (Figures 4D5C). The sorghum genome had three tandemly duplicated aromatic-L-amino acid decarboxylase genes (Figure 3B) and six PAL genes (Additional File 5: Figure S1), but the rice genome had two tandemly duplicated aromatic L-amino acid decarboxylase genes and four PAL genes [61], suggesting that the extra copy was acquired and thus strengthened the pathway after the sorghum–rice split. Cytochrome P450 domain–containing genes, which are often involved in phytoalexin synthesis and the scavenging of toxins, are abundant in sorghum, which has 326 such genes, versus 228 in rice [17], even though the target products have not been not fully identified *in vivo*. These duplications in sorghum have likely resulted in the diversity of both their genomic sequences and their expression; these genes have thereby developed different functions on an evolutionary time scale.

### Advantage of mRNA-seq for identification of pathogen-inducible genes

mRNA-seq provides information on all transcribed genes without the need to rely on annotation. Whole-genome tiling arrays can also be used to identify unannotated transcripts, but not for alternative splicing variants; this is the advantage of mRNA-seq over microarray technology. We predicted transcripts on the basis of the piling-up of mapped reads; 7674 transcripts were unannotated in Phytozome (Figure 2A). The differentially expressed unannotated transcripts encoded, for example, proteins similar to DFR, responsible for 3-deoxyanthocyanidin biosynthesis, or to maize *ZRP4 *[27], which encodes the *o*-methyltransferase involved in suberin biosynthesis (Figure 2B; Additional File 3: Table S3). Suberin is a component of the polymer matrices in lipophilic cell wall barriers. These barriers control the fluxes of gases, water, and solutes, and they also help to protect plants from biotic and abiotic stresses and to control plant morphology [62]. The unannotated differentially expressed genes could be identified only by mRNA-seq. Moreover, mRNA-seq could identify and distinguish the expression of each duplicated gene; it is therefore a powerful tool for analyzing genomes that have large numbers of such duplications. This application of mRNA-seq has generated many new leads and hypotheses in regard to metabolic pathways. Functional linkage of the transcriptome and metabolome is very important and should be elucidated systematically in the future.

Minimizing the technical error is important. We previously validated our sequence-based gene expression profiling against array-based technology in rice. For each gene from shoots (*N* = 14,575) and roots (*N* = 14,861), the ratio obtained from the array and the corresponding ratio obtained from RPKM were highly correlated over a broad range (*r* = 0.72 in shoot and 0.80 in root) [22]. Moreover, we confirmed the differential expression by using qRT-PCR of three biological replicates for the genes of interest (Additional file 6: Fig S3). We therefore consider that our sequence-based approach was generally valid as a gene expression profiling technology.

Following the rapid progress of massive parallel sequencing technology, whole mRNA sequencing has been used for gene expression profiling in sorghum. During the time when this paper was under review, a transcriptome analysis of *sorghum bicolor* in response to osmotic stress and abscisic acid was reported [63].

### Conslusions

Pathogen infection activated the glyoxylate shunt in the TCA cycle; this changes the role of the TCA cycle from energy production to synthesis of cell components. Genes encoding amino acid metabolizing enzymes located at the branch point between primary and secondary metabolism of phytoalexin synthesis or of sulfur-dependent detoxification were upregulated. The coordinated gene expression upon pathogen infection suggests the accumulation of the sorghum-specific phytochemicals 3-deoxyanthocyanidin. Particular genes in tandemly duplicated putative paralogs were highly upregulated. Key enzymes for synthesizing these sorghum-specific phytochemicals were not found in the corresponding region of the rice genome. Therefore, pathogen infection dramatically changed the expression of particular paralogs that putatively encode enzymes involved in the sorghum-specific metabolic network.

## Methods

### Plant materials and infection with target leaf spot

BTx623, a sorghum (*S. bicolor* (L.) Moench) cultivar susceptible to target leaf spot, was infected with *B. sorghicola* isolate BC-24 (Ministry of Agriculture, Forestry and Fisheries (MAFF) number 511379). The BC-24 strain was grown on vegetable juice (Campbell V8) agar for 10 days in the dark at 25°C and then placed under UV light for 10 days to induce conidial development. Conidia were harvested in 0.01% Tween-20, and the concentration of the suspension was adjusted to 4 × 10^5^ conidia/mL. At the 7- or 8-leaf stage, the sorghum plants were sprayed with 5 mL of the suspension per pot and then placed in 1/10000-a Wagner pots. The inoculated plants were kept in a moist chamber in the dark at 25°C for 16 h and then transferred to a greenhouse at 28.5 to 30°C. Seven days after inoculation, the plants were frozen in liquid nitrogen for RNA extraction.

### mRNA sequencing

For RNA extraction from each plant tissue, at least 5 biological replicates were collected, immediately frozen in liquid nitrogen, and mixed, to minimize the effect of transcriptome unevenness among plants. Total RNA was extracted by using an RNeasy Plant kit (Qiagen, Hilden, Germany). RNA quality was calculated with a Bioanalyzer 2100 algorithm (Agilent Technologies, Palo Alto, CA, USA); high-quality (RNA Integrity Number >8) RNA was used. Total RNA samples (10 μg) were subjected to cDNA construction for Illumina sequencing, in accordance with the protocol for the mRNA-Seq sample preparation kit (Illumina, San Diego, CA, USA). Oligo(dT) magnetic beads were used to isolate poly(A) RNA from the total RNA samples. The mRNA was fragmented by being heated at 94°C for 5 min. First-strand cDNA was synthesized using random hexamer primers for 25°C/10 min, 42°C/50 min, and 70°C/15 min. After the first strand had been synthesized, dNTPs, RNaseH, and DNA polymerase I were added to synthesize second-strand DNA for 2.5 h at 16°C. The ends of double-stranded cDNA were repaired by using T4 DNA polymerase and Klenow DNA polymerase and phosphorylated by using T4 polynucleotide kinase. A single “A” base was added to the cDNA molecules by using Klenow exonuclease, and the fragments were ligated to the Paired End (PE) adapters from the Illumina mRNA-Seq kit. cDNA having 200- ± 25-bp fragments were collected. The purified cDNA was amplified by 15 cycles of PCR for 98°C/10 s, 65°C/30 s, and 72°C/30 s using PE1.0 and PE2.0 primers.

### Constructing gene models and searching for homology to genes encoding known proteins

cDNA was sequenced (single read) by using an Illumina Genome Analyzer IIx. Data on two technical replicates (two sequencing lanes of a cDNA sample from mock- or pathogen-infected leaf, corresponding to about 28.7 to 34.6 million 76-bp reads) were accumulated. The default Illumina pipeline quality filter, which uses a threshold of CHASTITY ≥ 0.6, was used to identify clusters with low signal-to-noise ratios. CHASTITY is defined as “the ratio of the highest of the four (base-type) intensities to the sum of the highest two.” Passed filter reads were mapped onto the sorghum reference genome by using Bowtie (0.12.7) [23], and TopHat (1.1.4) [24] with the following options: segment length, 25; minimum intron length, 30; maximum intron length, 6000; maximum multihits, 40; number of threads: 2. Cufflinks (0.9.3) [25] was used for prediction of genes by the piling-up of mapped reads. Unannotated transcripts were screened by comparison with Phytozome annotation (Sbicolor_79). To predict the functions of the unannotated transcripts, BLASTx searches were performed against Uniprot (Rel. 2011_01) and RefSeq (release 45) (identity ≥30% and coverage ≥30%). Members of gene families in the sorghum genome were grouped on the basis of amino acid sequence similarity. Homologous genes in the rice genome were identified on the basis of synteny between sorghum and rice by using RAP-DB (GBrowse_syn) [61]. Genes differentially expressed (up or down) in control and infected tissues were identified by G-test (FDR <0.01). Highly upregulated genes were mapped on metabolic maps by using the KEGG (Kyoto Encyclopedia of Genes and Genomes) database [64].

### Quantitative RT-PCR (qRT-PCR)

For RNA extraction from each plant tissue, three biological replicates were collected independently, immediately frozen in liquid nitrogen. One microgram of total RNA was reverse-transcribed in a 20-μL reaction mixture from a Transcriptor First Strand cDNA Synthesis Kit (Roche, Basel, Switzerland). qRT-PCR was performed in a 20-μL reaction mixture containing 2 × SYBR Master Mix (Kapa), 1 μL of cDNA template (1:10 dilution), and newly designed primers for each gene of interest (Additional file 7: Table S5). qRT-PCR of three biological replicates for each sample was performed using a LightCycler 480 System with its relative quantification software (ver. 1.2) based on the delta-delta-Ct method (Roche). qRT-PCR was performed for 10 s at 95°C, 5 s at 55°C, and 10 s at 72°C. The expression level for each reaction was normalized against the expression level of the actin gene [65].

### Thin layer chromatography (TLC)

Leaf samples infected by *Bipolaris sorghicola* were harvested, and the pigments were extracted by incubation overnight at 4°C with methanol containing 0.1% HCl. The extracted 3-deoxyanthocyanins were hydrolyzed with 1 N HCl at 100°C for 1 h [66]. Aglycones were extracted with isoamyl alcohol, then dried and dissolved in methanol containing 0.1% HCl. Anthocyanidin aglycones were developed on TLC Cellulose F plates (Merck, Darmstadt, Germany) using HCl:AcOH:water (3:30:60 v/v/v) as the solvent. Chemically synthesized apigeninidin (Fluka Sigma-Aldrich, St. Louis, MO, USA) was used as a standard.

### Accession Number

All primary sequence read data have been submitted to DDBJ (DNA Data Bank of Japan) Sequence Read Archive [DRA000387].

## Authors’ contributions

H. Kaw., JO, and H. Miz. prepared plant materials and performed cDNA synthesis and qRT-PCR; H. Kan. and H. Min. performed sequencing experiments and primary data analysis; YK and TI performed the data analysis; H. Miz., Y. Kaw. , and TM designed the study; H. Miz. wrote the manuscript. All authors read and approved the final manuscript.

## Supplementary Material

Additional file 1 **Table S1.** Expression ratios and ORF predictions of Phytozome transcripts. Click here for file

Additional file 2 **Table S2.** Expression ratios and ORF predictions of unannotated transcripts. Click here for file

Additional file 3 **Table S3.** Examples of differentially expressed novel transcripts. Click here for file

Additional file 4 **Table S4.** Examples of expression ratios and ORF predictions of differentially expressed transcripts. Click here for file

Additional file 5 **Figure S1.** Expression of genes associated with secondary metabolism of phenylalanine to naringenin. RPKMs of phenylalanine ammonia lyase (PAL), trans-cinnamate 4-monooxygenase (C4H), 4-coumarate:CoA ligase (4CL), chalcone synthase (CHS), and chalcone isomerase (CHI) are shown. PAL and CHS had tandemly duplicated putative paralogs. Figure S2. Expression of genes associated with target leaf spot infection. RPKMs of F3′Hs, ds1 LRR-RK, polyphenol oxidase, and C4 photosynthesis genes are shown. F3′H and polyphenol oxidases had tandemly duplicated putative paralogs that were differentially expressed. Click here for file

Additional file 6 **Figure S3.** Validation of expression level by quantitative real-time PCR (qRT-PCR). qRT-PCR of three biological replicates for each sample was performed and the means and standard deviations are shown. The expression level for each reaction was normalized against the expression level of the actin gene. Click here for file

Additional file 7 **Table S5.** Primers used. Click here for file

## References

[B1] Kuc J Phytoalexins, Stress Metabolism, and Disease Resistance in Plants Annual Review of Phytopathology 1995 33 275 297 10.1146/annurev.py.33.090195.001423 18999962

[B2] Snyder BA Nicholson RL Synthesis of phytoalexins in sorghum as a site-specific response to fungal ingress Science 1990 248 4963 1637 1639 10.1126/science.248.4963.1637 17746504

[B3] Graham TL Flavonoid and flavonol glycoside metabolism in Arabidopsis Plant PhysiolBioch 1998 36 1–2 135 144

[B4] Boudet AM Lignins and lignification: Selected issues Plant Physiol Bioch 2000 38 1–2 81 96

[B5] Vogt T Phenylpropanoid Biosynthesis Molecular Plant 2010 3 1 2 20 10.1093/mp/ssp106 20035037

[B6] Mizutani M Ohta D Diversification of P450 genes during land plant evolution Annu Rev Plant Biol 2010 61 291 315 10.1146/annurev-arplant-042809-112305 20192745

[B7] Powles SB Yu Q Evolution in action: plants resistant to herbicides Annu Rev Plant Biol 2010 61 317 347 10.1146/annurev-arplant-042809-112119 20192743

[B8] Lo SCC De Verdier K Nicholson RL Accumulation of 3-deoxyanthocyanidin phytoalexins and resistance to Colletotrichum sublineolum in sorghum Physiol Mol Plant P 1999 55 5 263 273 10.1006/pmpp.1999.0231

[B9] Nielsen KA Tattersall DB Jones PR Moller BL Metabolon formation in dhurrin biosynthesis Phytochemistry 2008 69 1 88 98 10.1016/j.phytochem.2007.06.033 17706731

[B10] Wittstock U Gershenzon J Constitutive plant toxins and their role in defense against herbivores and pathogens Curr Opin Plant Biol 2002 5 4 300 307 10.1016/S1369-5266(02)00264-9 12179963

[B11] Tattersall DB Bak S Jones PR Olsen CE Nielsen JK Hansen ML Hoj PB Moller BL Resistance to an herbivore through engineered cyanogenic glucoside synthesis Science 2001 293 5536 1826 1828 10.1126/science.1062249 11474068

[B12] Selmar D Irandoost Z Wray V Dhurrin-6'-glucoside, a cyanogenic diglucoside from Sorghum bicolor Phytochemistry 1996 43 3 569 572 10.1016/0031-9422(96)00297-X 8987580

[B13] Busk PK Moller BL Dhurrin synthesis in sorghum is regulated at the transcriptional level and induced by nitrogen fertilization in older plants Plant Physiol 2002 129 3 1222 1231 10.1104/pp.000687 12114576 PMC166516

[B14] Czarnota MA Paul RN Dayan FE Nimbal CI Weston LA Mode of action, localization of production, chemical nature, and activity of sorgoleone: A potent PSII inhibitor in Sorghum spp. root exudates Weed Technol 2001 15 4 813 825 10.1614/0890-037X(2001)015[0813:MOALOP]2.0.CO;2

[B15] Zummo N Gourley LM Occurrence of Target Leaf-Spot (Bipolaris-Sorghicola) on Sorghum in Mississippi Plant Dis 1987 71 11 1045 1045

[B16] Kawahigashi H Kasuga S Ando T Kanamori H Wu J Yonemaru J Sazuka T Matsumoto T Positional cloning of ds1, the target leaf spot resistance gene against Bipolaris sorghicola in sorghum Theor Appl Genet 2011 123 1 131 142 10.1007/s00122-011-1572-1 21442410

[B17] Paterson AH Bowers JE Bruggmann R Dubchak I Grimwood J Gundlach H Haberer G Hellsten U Mitros T Poliakov A The Sorghum bicolor genome and the diversification of grasses Nature 2009 457 7229 551 556 10.1038/nature07723 19189423

[B18] Sugarbaker DJ Richards WG Gordon GJ Dong L De Rienzo A Maulik G Glickman JN Chirieac LR Hartman ML Taillon BE Transcriptome sequencing of malignant pleural mesothelioma tumors Proc Natl Acad Sci U S A 2008 105 9 3521 3526 10.1073/pnas.0712399105 18303113 PMC2265129

[B19] Torres TT Metta M Ottenwalder B Schlotterer C Gene expression profiling by massively parallel sequencing Genome Res 2008 18 1 172 177 18032722 10.1101/gr.6984908 PMC2134766

[B20] Pepke S Wold B Mortazavi A Computation for ChIP-seq and RNA-seq studies Nat Methods 2009 6 11 Suppl S22 32 19844228 10.1038/nmeth.1371 PMC4121056

[B21] Wang Z Gerstein M Snyder M RNA-Seq: a revolutionary tool for transcriptomics Nat Rev Genet 2009 10 1 57 63 10.1038/nrg2484 19015660 PMC2949280

[B22] Mizuno H Kawahara Y Sakai H Kanamori H Wakimoto H Yamagata H Oono Y Wu J Ikawa H Itoh T Massive parallel sequencing of mRNA in identification of unannotated salinity stress-inducible transcripts in rice (Oryza sativa L.) BMC Genomics 2010 11 683 10.1186/1471-2164-11-683 21122150 PMC3016417

[B23] Langmead B Trapnell C Pop M Salzberg SL Ultrafast and memory-efficient alignment of short DNA sequences to the human genome Genome Biol 2009 10 3 R25 10.1186/gb-2009-10-3-r25 19261174 PMC2690996

[B24] Trapnell C Pachter L Salzberg SL TopHat: discovering splice junctions with RNA-Seq Bioinformatics 2009 25 9 1105 1111 10.1093/bioinformatics/btp120 19289445 PMC2672628

[B25] Trapnell C Williams BA Pertea G Mortazavi A Kwan G van Baren MJ Salzberg SL Wold BJ Pachter L Transcript assembly and quantification by RNA-Seq reveals unannotated transcripts and isoform switching during cell differentiation Nat Biotechnol 2010 28 5 511 515 10.1038/nbt.1621 20436464 PMC3146043

[B26] Mortazavi A Williams BA McCue K Schaeffer L Wold B Mapping and quantifying mammalian transcriptomes by RNA-Seq Nat Methods 2008 5 7 621 628 10.1038/nmeth.1226 18516045 PMC13303166

[B27] Held BM Wang H John I Wurtele ES Colbert JT An mRNA putatively coding for an O-methyltransferase accumulates preferentially in maize roots and is located predominantly in the region of the endodermis Plant Physiol 1993 102 3 1001 1008 10.1104/pp.102.3.1001 8278520 PMC158874

[B28] Liu H Du Y Chu H Shih CH Wong YW Wang M Chu IK Tao Y Lo C Molecular dissection of the pathogen-inducible 3-deoxyanthocyanidin biosynthesis pathway in sorghum Plant Cell Physiol 2010 51 7 1173 1185 10.1093/pcp/pcq080 20529887

[B29] Adamkova S Luhova L Petrivalsky M Pec P Characterization of enzyme phenylalanine ammonia-lyase and its role in activation of defensive mechanisms in plants Chem Listy 2006 100 7 486 494

[B30] Dayan FE Howell J Weidenhamer JD Dynamic root exudation of sorgoleone and its in planta mechanism of action J Exp Bot 2009 60 7 2107 2117 10.1093/jxb/erp082 19357432 PMC2682501

[B31] Xie DY Sharma SB Paiva NL Ferreira D Dixon RA Role of anthocyanidin reductase, encoded by BANYULS in plant flavonoid biosynthesis Science 2003 299 5605 396 399 10.1126/science.1078540 12532018

[B32] Winkel BS Metabolic channeling in plants Annu Rev Plant Biol 2004 55 85 107 10.1146/annurev.arplant.55.031903.141714 15725058

[B33] Du Y Chu H Wang M Chu IK Lo C Identification of flavone phytoalexins and a pathogen-inducible flavone synthase II gene (SbFNSII) in sorghum J Exp Bot 2010 61 4 983 994 10.1093/jxb/erp364 20007684 PMC2826645

[B34] Czarnota MA Paul RN Weston LA Duke SO Anatomy of sorgoleone-secreting root hairs of Sorghum species Int J Plant Sci 2003 164 6 861 866 10.1086/378661

[B35] Baerson SR Dayan FE Rimando AM Nanayakkara NPD Liu CJ Schroerder J Fishbein M Pan Z Kagan IA Pratt LH A functional genomics investigation of allelochemical biosynthesis in Sorghum bicolor root hairs Journal of Biological Chemistry 2008 283 6 3231 3247 17998204 10.1074/jbc.M706587200

[B36] Dayan FE Rimando AM Pan ZQ Baerson SR Gimsing AL Duke SO Sorgoleone Phytochemistry 2010 71 10 1032 1039 10.1016/j.phytochem.2010.03.011 20385394

[B37] Cook D Rimando AM Clemente TE Schroder J Dayan FE Nanayakkara NP Pan Z Noonan BP Fishbein M Abe I Alkylresorcinol synthases expressed in Sorghum bicolor root hairs play an essential role in the biosynthesis of the allelopathic benzoquinone sorgoleone Plant Cell 2010 22 3 867 887 10.1105/tpc.109.072397 20348430 PMC2861460

[B38] Cook D Rimando AM Clemente TE Schroder J Dayan FE Nanayakkara NPD Pan ZQ Noonan BP Fishbein M Abe I Alkylresorcinol Synthases Expressed in Sorghum bicolor Root Hairs Play an Essential Role in the Biosynthesis of the Allelopathic Benzoquinone Sorgoleone Plant Cell 2010 22 3 867 887 10.1105/tpc.109.072397 20348430 PMC2861460

[B39] Dunn MF Ramirez-Trujillo JA Hernandez-Lucas I Major roles of isocitrate lyase and malate synthase in bacterial and fungal pathogenesis Microbiology 2009 155 Pt 10 3166 3175 19684068 10.1099/mic.0.030858-0

[B40] Eastmond PJ Graham IA Re-examining the role of the glyoxylate cycle in oilseeds Trends Plant Sci 2001 6 2 72 78 10.1016/S1360-1385(00)01835-5 11173291

[B41] Facchini PJ Huber-Allanach KL Tari LW Plant aromatic L-amino acid decarboxylases: evolution, biochemistry, regulation, and metabolic engineering applications Phytochemistry 2000 54 2 121 138 10.1016/S0031-9422(00)00050-9 10872203

[B42] Beinert H A tribute to sulfur European Journal of Biochemistry 2000 267 18 5657 5664 10.1046/j.1432-1327.2000.01637.x 10971575

[B43] Noctor G Gomez L Vanacker H Foyer CH Interactions between biosynthesis, compartmentation and transport in the control of glutathione homeostasis and signalling Journal of Experimental Botany 2002 53 372 1283 1304 10.1093/jexbot/53.372.1283 11997376

[B44] Watanabe M Mochida K Kato T Tabata S Yoshimoto N Noji M Saito K Comparative Genomics and Reverse Genetics Analysis Reveal Indispensable Functions of the Serine Acetyltransferase Gene Family in Arabidopsis Plant Cell 2008 20 9 2484 2496 10.1105/tpc.108.060335 18776059 PMC2570737

[B45] Hermsmeier D Schittko U Baldwin IT Molecular interactions between the specialist herbivore Manduca sexta (Lepidoptera, Sphingidae) and its natural host Nicotiana attenuata I. Large-scale changes in the accumulation of growth- and defense-related plant mRNAs Plant Physiol 2001 125 2 683 700 10.1104/pp.125.2.683 11161026 PMC64870

[B46] Kombrink E Hahlbrock K Rapid, Systemic Repression of the Synthesis of Ribulose 1,5-Bisphosphate Carboxylase Small-Subunit Messenger-Rna in Fungus-Infected or Elicitor-Treated Potato Leaves Planta 1990 181 2 216 219 10.1007/BF02411541 24196739

[B47] Aguero ME Gevens A Nicholson RL Interaction of Cochliobolus heterostrophus with phytoalexin inclusions in Sorghum bicolor Physiol Mol Plant P 2002 61 5 267 271 10.1006/pmpp.2003.0440

[B48] Boddu J Svabek C Ibraheem F Jones AD Chopra S Characterization of a deletion allele of a sorghum Myb gene, yellow seed1 showing loss of 3-deoxyflavonoids Plant Science 2005 169 3 542 552 10.1016/j.plantsci.2005.05.007

[B49] Ibraheem F Gaffoor I Chopra S Flavonoid phytoalexin-dependent resistance to anthracnose leaf blight requires a functional yellow seed1 in Sorghum bicolor Genetics 2010 184 4 915 926 10.1534/genetics.109.111831 20083611 PMC2865927

[B50] Grotewold E Chamberlin M Snook M Siame B Butler L Swenson J Maddock S Clair GS Bowen B Engineering secondary metabolism in maize cells by ectopic expression of transcription factors Plant Cell 1998 10 5 721 740 9596632 PMC144024

[B51] Rushton PJ Somssich IE Ringler P Shen QJ WRKY transcription factors Trends Plant Sci 2010 15 5 247 258 10.1016/j.tplants.2010.02.006 20304701

[B52] Zhang Y Wang L The WRKY transcription factor superfamily: its origin in eukaryotes and expansion in plants BMC Evol Biol 2005 5 1 1 10.1186/1471-2148-5-1 15629062 PMC544883

[B53] Bough WA Gander JE Exogenous L-Tyrosine Metabolism and Dhurrin Turnover in Sorghum Seedlings Phytochemistry 1971 10 1 67 77 10.1016/S0031-9422(00)90252-8

[B54] Adewusi SRA Turnover of Dhurrin in Green Sorghum Seedlings Plant Physiology 1990 94 3 1219 1224 10.1104/pp.94.3.1219 16667820 PMC1077365

[B55] Lieberei R Biehl B Giesemann A Junqueira NTV Cyanogenesis Inhibits Active Defense Reactions in Plants Plant Physiology 1989 90 1 33 36 10.1104/pp.90.1.33 16666758 PMC1061671

[B56] Zhu-Salzman K Salzman RA Ahn JE Koiwa H Transcriptional regulation of sorghum defense determinants against a phloem-feeding aphid Plant Physiol 2004 134 1 420 431 10.1104/pp.103.028324 14701914 PMC316321

[B57] IRGSP The map-based sequence of the rice genome Nature 2005 436 7052 793 800 10.1038/nature03895 16100779

[B58] Bowers JE Abbey C Anderson S Chang C Draye X Hoppe AH Jessup R Lemke C Lennington J Li Z A high-density genetic recombination map of sequence-tagged sites for sorghum, as a framework for comparative structural and evolutionary genomics of tropical grains and grasses Genetics 2003 165 1 367 386 14504243 10.1093/genetics/165.1.367 PMC1462765

[B59] Kim JS Klein PE Klein RR Price HJ Mullet JE Stelly DM Chromosome identification and nomenclature of Sorghum bicolor Genetics 2005 169 2 1169 1173 10.1534/genetics.104.035980 15489512 PMC1449135

[B60] Bowers JE Arias MA Asher R Avise JA Ball RT Brewer GA Buss RW Chen AH Edwards TM Estill JC Comparative physical mapping links conservation of microsynteny to chromosome structure and recombination in grasses Proc Natl Acad Sci U S A 2005 102 37 13206 13211 10.1073/pnas.0502365102 16141333 PMC1201573

[B61] Rice_Annotation_Project Curated genome annotation of Oryza sativa ssp. japonica and comparative genome analysis with Arabidopsis thaliana Genome Res 2007 17 2 175 183 10.1101/gr.5509507 17210932 PMC1781349

[B62] Pollard M Beisson F Li Y Ohlrogge JB Building lipid barriers: biosynthesis of cutin and suberin Trends Plant Sci 2008 13 5 236 246 10.1016/j.tplants.2008.03.003 18440267

[B63] Dugas DV Monaco MK Olsen A Klein RR Kumari S Ware D Klein PE Functional annotation of the transcriptome of Sorghum bicolor in response to osmotic stress and abscisic acid BMC Genomics 2011 12 514 10.1186/1471-2164-12-514 22008187 PMC3219791

[B64] Kanehisa M Goto S KEGG: Kyoto Encyclopedia of Genes and Genomes Nucleic Acids Research 2000 28 1 27 30 10.1093/nar/28.1.27 10592173 PMC102409

[B65] Shih CH Chu IK Yip WK Lo C Differential expression of two flavonoid 3'-hydroxylase cDNAs involved in biosynthesis of anthocyanin pigments and 3-deoxyanthocyanidin phytoalexins in sorghum Plant Cell Physiol 2006 47 10 1412 1419 10.1093/pcp/pcl003 16943219

[B66] Shimizu T Nakamura M Kato Y Yoshihara K Sawai J Terahara N Structural determination, stability, and antimicrobial activity of Sorghum nervosum seed coat pigments (Kaoliang color) Jpn J Food Chem 1996 3 21 26

