# Supplementary figures and images for: Global transcriptome analysis reveals distinct expression among duplicated genes during sorghum-interaction
Source: BMC Plant Biol. 2012 Jul 29;12:121. doi: 10.1186/1471-2229-12-121 (PMC3480847; doi:10.1186/1471-2229-12-121)

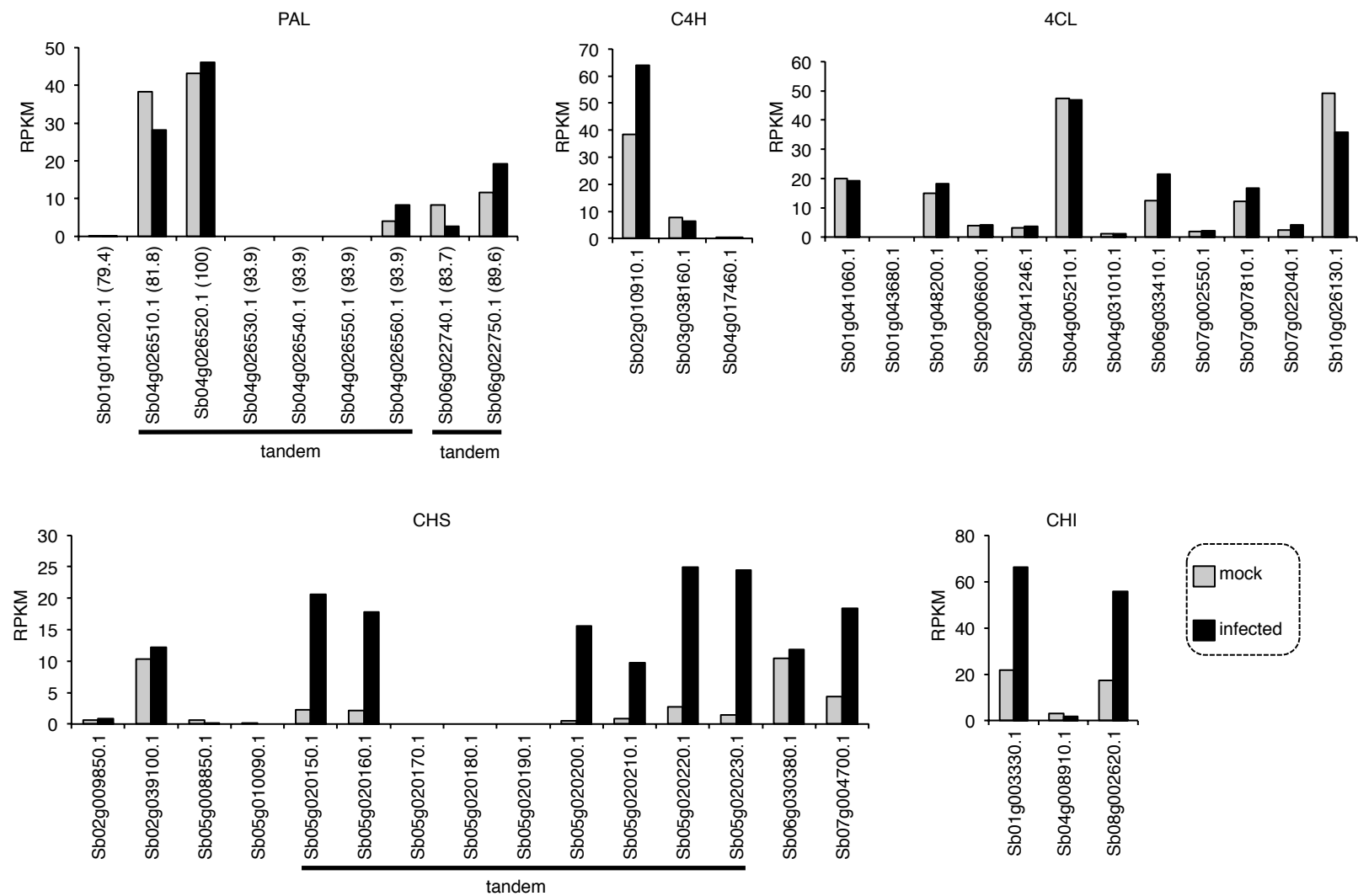

Figure S1

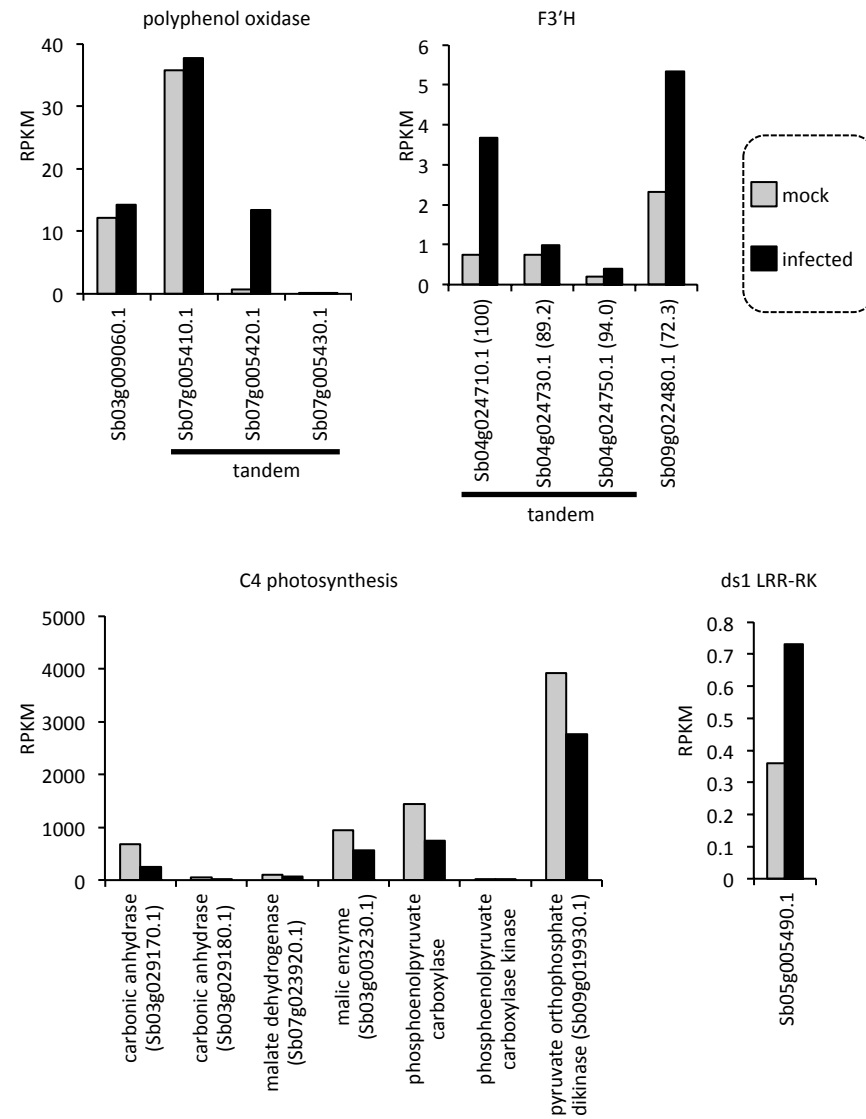

Figure S2

Supplement: Additional file 5 — Figure S1. Expression of genes associated with secondary metabolism of phenylalanine to naringenin. RPKMs of phenylalanine ammonia lyase (PAL), trans-cinnamate 4-monooxygenase (C4H), 4-coumarate:CoA ligase (4CL), chalcone synthase (CHS), and chalcone isomerase (CHI) are shown. PAL and CHS had tandemly duplicated putative paralogs. Figure S2. Expression of genes associated with target leaf spot infection. RPKMs of F3′Hs, ds1 LRR-RK, polyphenol oxidase, and C4 photosynthesis genes are shown. F3′H and polyphenol oxidases had tandemly duplicated putative paralogs that were differentially expressed. [file 1471-2229-12-121-S5.pdf]

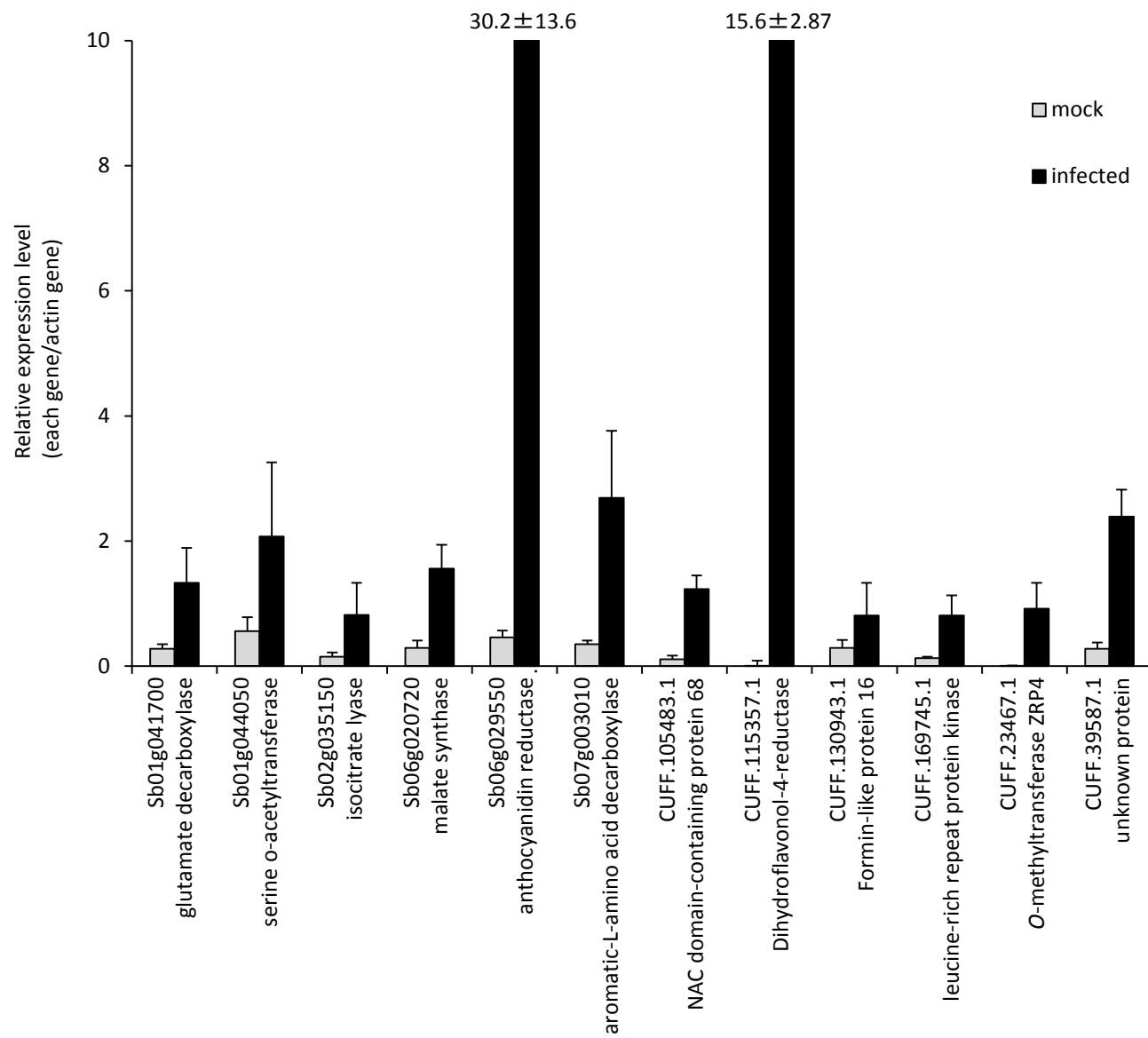

Figure S3

Supplement: Additional file 6 — Figure S3. Validation of expression level by quantitative real-time PCR (qRT-PCR). qRT-PCR of three biological replicates for each sample was performed and the means and standard deviations are shown. The expression level for each reaction was normalized against the expression level of the actin gene. [file 1471-2229-12-121-S6.pdf]
